# Biocontrol and plant growth-promoting activity of rhizobacteria from Chinese fields with contaminated soils

**DOI:** 10.1111/1751-7915.12158

**Published:** 2014-09-15

**Authors:** Xuefei Wang, Dmitri V Mavrodi, Linfeng Ke, Olga V Mavrodi, Mingming Yang, Linda S Thomashow, Na Zheng, David M Weller, Jibin Zhang

**Affiliations:** 1 State Key Laboratory of Agricultural Microbiology, College of Life Science and Technology, Huazhong Agricultural UniversityWuhan, Hubei, 430070, China; 2 Department of Plant Pathology, Washington State UniversityPullman, WA, 99164-6430, USA; 3 Department of Biological Sciences, The University of Southern MississippiHattiesburg, MS, 39406, USA; 4 Agricultural Research Service, Root Disease and Biological Control Research Unit, United States Department of AgriculturePullman, WA, 99164-6430, USA

## Abstract

The aim of this study was to inventory the types of plant growth-promoting rhizobacteria (PGPR) present in the rhizosphere of plants grown in soils contaminated with heavy metals, recalcitrant organics, petroleum sewage or salinity in China. We screened 1223 isolates for antifungal activity and about 24% inhibited *Rhizoctonia solani* or *Sclerotinia sclerotiorum*. Twenty-four strains inhibitory to *R. solani*, *Gaeumannomyces graminis* var. *tritici* and/or *S. sclerotiorum* and representing the dominant morphotypes were assayed for PGPR activity. Seven strains contained *phlD*, *prnD*, *pltC* or *phzF* genes and produced the antibiotics 2,4-diacetylphloroglucinol, pyrrolnitrin, pyoluteorin and phenazines respectively. Six strains contained *acdS*, which encodes 1-aminocyclopropane-1-carboxylic acid deaminase. Phylogenetic analysis of 16S rDNA and *phlD*, *phzF* and *acdS* genes demonstrated that some strains identified as *Pseudomonas* were similar to model PGPR strains *Pseudomonas protegens* Pf-5, *Pseudomonas chlororaphis* subsp. *aureofaciens* 30–84 and *P. brassicacearum* Q8r1-96. *Pseudomonas protegens-* and *P. chlororaphis-*like strains had the greatest biocontrol activity against Rhizoctonia root rot and take-all of wheat. *Pseudomonas protegens* and *P. brassicacearum-*like strains showed the greatest promotion of canola growth. Our results indicate that strains from contaminated soils are similar to well-described PGPR found in agricultural soils worldwide.

Growth-promoting rhizobacteria in polluted soils

## Introduction

Soil salinization and contamination by organic compounds (i.e. petroleum products and solvents) and heavy metals (i.e. Cd, Cr, Cu, Hg, Pb) are important environmental problems worldwide and have negative impacts on both human health and agricultural productivity (Jing *et al*., 2007). Soil pollution is of particular concern in developing countries where rapid industrialization has led to contamination of agricultural lands, especially in small farms that are sources of food for consumption by local populations. In a survey of contamination levels of heavy metals in agricultural soils in China, metal concentrations of Cd were significantly higher than in non-contaminated soils (Wei and Yang, 2010). Similarly, because of the accumulation of mine tailing outfalls, the content of Cu in the wetlands of Daye County, Hubei province, was 18 times higher than the background value (Guo *et al*., 2011). The build-up of pollutants in soils and their subsequent leaching into the ecosystem has cascading effects, adversely impacting plant growth and yield (Cheng, 2003b; Huang *et al*., 2005; Parida and Das, 2005). The uptake of soil pollutants by crop plants and the resultant entry of carcinogens, endocrine disruptors and neurotoxins into the food chain are major concerns for human and animal health (Cheng, 2003a; Cai *et al*., 2009). In addition, soil pollutants can positively or negatively influence crop susceptibility to pathogens and insects (Elmer, 2002; Poschenrieder *et al*., 2006). For example, salinity can predispose plants to infection by soilborne pathogens or increase the incidence and severity of disease (Triky-Dotan *et al*., 2005; Roubtsova and Bostock, 2009). In contrast, exposure of wheat to a mild dose of cadmium induced resistance against *Fusarium* infection (Mittra *et al*., 2004).

Rhizobacteria are able to colonize and persist on roots and some, known as plant growth-promoting rhizobacteria (PGPR), are able to promote plant growth (Weller, 2007). PGPR improve plant growth directly by supplying nutrients (e.g. phosphorous, nitrogen), producing phytohormones [e.g. indoleacetic acid (IAA)], and/or decreasing the level of ethylene that is produced when the plant is stressed (Li *et al*., 2007; Lugtenberg and Kamilova, 2009). PGPR also antagonize plant pathogens and/or induce systemic resistance in the plant to disease (Weller *et al*., 2007; Lugtenberg and Kamilova, 2009). Because plants lack effector-triggered immunity to many soilborne pathogens, they rely on PGPR for defence against pathogen attack (Weller *et al*., 2007).

Soil pollution, especially by heavy metals, can also decrease the metabolic activity, biomass and diversity of microorganisms in the bulk soil and the rhizosphere (Sandaa *et al*., 1999; Gremion *et al*., 2004; Jing *et al*., 2007) and limit the effectiveness of PGPR to protect roots against disease. However, PGPR with metal resistance and the ability to mitigate the toxic effects of soil pollutants are present in polluted soils (Huang *et al*., 2005; Reed and Glick, 2005; Jing *et al*., 2007). For example, *Pseudomonas asplenii* AC isolated from polycyclic aromatic hydrocarbon-contaminated soils significantly increased the root and shoot growth of canola in copper and creosote-contaminated soils (Reed and Glick, 2005). Salt-tolerant *Pseudomonas chlororaphis* TSAU13, isolated from the rhizosphere of wheat grown in a salinated soil (Egamberdieva and Kucharova, 2009), improved the shoot and root growth of common bean grown in soil amended with NaCl and protected cucumber and tomato against *Fusarium solani* (Egamberdieva, 2011).

Despite progress in understanding the crucial role of PGPR in helping plants adapt to environmental stress, major gaps remain in our knowledge of how soil pollution affects the population levels, diversity and activity of PGPR. Our study is part of an ongoing cooperative project in China and the United States with the goal of identifying new PGPR and characterizing their role in the promotion of soil and plant health. Our study focused on rhizobacteria isolated from Chinese soils polluted with heavy metals, recalcitrant organic compounds, petroleum sewage or excessive salinization. We have hypothesized that certain groups of PGPR and strains harbouring biocontrol and growth-promoting genes are cosmopolitan and conserved in soil microbiomes in agroecosystems worldwide regardless of the condition of the soil, and that plants enrich and support those PGPR best adapted to help them grow and resist diseases and other pests. Nothing is known about the composition of PGPR in soils from these polluted sites, thus making them ideal to test our hypothesis.

Our specific objectives were to: (i) isolate and identify PGPR, (ii) determine their biocontrol activity against take-all disease and Rhizoctonia root rot on wheat, (iii) determine their growth-promoting activity on rapeseed/canola, and (iv) characterize their growth-promoting traits and genes. Wheat and rapeseed/canola were selected for our study because China is the world's largest producer of both of these crops and they are often grown in rotation. Rhizoctonia root rot caused by *Rhizoctonia solani* AG-8 and take-all caused by *Gaeumannomyces graminis* var. *tritici* are two important soilborne diseases worldwide, against which wheat has no resistance (Cook *et al*., 2002; Paulitz *et al*., 2002; Yang *et al*., 2011); however, biocontrol with PGPR is a viable approach to controlling them. Poor emergence is a chronic problem in rapeseed/canola production in both China and the United States and application of PGPR as seed treatments can greatly enhance shoot and root growth.

Here we report that strains of well-described PGPR species were among the common morphotypes from the rhizosphere of wheat and rapeseed grown in highly polluted soils in China. Strains of *Lysobacter capsici*, *P. chlororaphis*, *Pseudomonas protegens* and those related to *P. brassicacearum* had some of the greatest biocontrol and/or growth promoting activity. PGPR genes in these morphotypes included *phlD*, *phzF*, *prnD*, *pltC* and *acdS*, and traits included the ability to produce 2,4-diacetylphloroglucinol (2,4-DAPG), phenazines, pyrrolnitrin, pyoluteorin, cellulase, siderophores, protease and IAA.

## Results

### Population densities and antagonistic activity of rhizobacteria from contaminated soils

The population sizes of total culturable aerobic, heterotrophic bacteria in the rhizosphere of wheat after three growth cycles in the greenhouse in the contaminated soils from five fields located in Hubei, Jiangxi and Jiangsu provinces of China ranged from 4.2 × 10^6^ to 3.4 × 10^8^ colony-forming units (cfu) g^−1^ fresh weight of root (Table 1). Populations recovered from rapeseed grown in the same soils ranged from 3.6 × 10^5^ to 1.3 × 10^6^ cfu g^−1^ fresh weight of root (Table 1). From the wheat and rapeseed rhizospheres, 1223 bacterial isolates (605 from wheat and 618 from rapeseed) were randomly selected from dilution plates of ^1^/_3_ × King's medium B (KMB) and ^1^/_10_ × tryptic soy agar (TSA). The percentage of isolates from wheat inhibitory to *R. solani* and from rapeseed inhibitory to *Sclerotinia sclerotiorum* varied among soils but averaged 24% and 23% respectively (Table 1). Isolates from the field contaminated with recalcitrant organic compounds in Hukou County, Jiangxi province were the least inhibitory to *R. solani* and *S. sclerotiorum* (6.7% and 15.1% respectively) as compared with isolates from the other soils.

**Table 1:** Population densities of total culturable aerobic, heterotrophic bacteria in the rhizosphere of wheat and rapeseed and ability of the bacteria to inhibit soilborne pathogens *in vitro*

		Wheat rhizosphere		Canola rhizosphere	
Location^a^	Soil contaminant	cfu g^−1^ fresh weight root	Inhibitory to *Rhizoctonia* AG-8 (%)	cfu g^−1^ fresh weight root	Inhibitory to *Sclerotinia sclerotiorum* (%)
Daye County	Heavy metals	5.48 ± 2.85 × 10^7^	27.2	1.34 ± 0.44 × 10^6^	15.5
Hukou County	Recalcitrant organic compounds	4.18 ± 3.0 × 10^6^	6.7	4.02 ± 1.41 × 10^5^	15.1
Jinmen City	Petroleum products	5.54 ± 2.3 × 10^7^	28.0	8.4 ± 0.35 × 10^5^	36.2
Qidong City (Dongyuan)	Salt	2.69 ± 1.4 × 10^8^	19.6	7.06 ± 0.13 × 10^5^	27.5
Qidong City (Yinyang)	Salt	3.39 ± 3.37 × 10^8^	37.2	3.56 ± 0.66 × 10^5^	21.8

a Soil samples were collected in Daye County and Jinmen City in Hubei province, Hukou county in Jiangxi province, and Qidong City near Dongyuan and Yinyang in Jiangsu province; GPS coordinates of these sites are 30°10′N, 114°56′E; 29°46′N, 116°15′E; 31°01′N, 112°13′E; 31°58′N, 121°46′E; and 31°44′N, 121°54′E respectively.

Crop and native plants and weeds were dug from the contaminated soil at each site, soil attached to the roots was removed, transported to Wuhan, and wheat or rapeseed was grown in the soil for three cycles. Population densities of total culturable, aerobic rhizosphere bacteria were determined by the end-point dilution assay and single colonies of bacteria were selected from plates of ^1^/_3_ × KMB or ^1^/_10_ × TSA at the end of the third growth cycle of wheat or rapeseed.

### Identity of the PGPR and their biocontrol and growth-promoting traits and genes

Twenty-four strains representative of the dominant morphotypes (Table 2) were screened for growth-promoting and biocontrol-related traits and genes. Of these 24 strains, 15, 11 and 20, respectively, inhibited the growth of *G. graminis* var. *tritici* ARS-A1, *R. solani* AG-8 C-1 and *S. sclerotiorum* MGSCF180002 (Table 3).

**Table 2:** Bacteria used in this study

Strain^a^	Source and type of contamination	References
*Bacillus* sp. TY5107	Qidong City (near Yinyang), Jiangsu Province, China (excessive salinization)	This study
*Delftia tsuruhatensis* TM3205	Jinmen City, Hubei Province, China (petroleum products)	This study
*Lysobacter capsici* TM5405	Qidong City (near Yinyang), Jiangsu Province, China (excessive salinization)	This study
*Lysobacter enzymogenes* TM2502	Hukou County, Jiangxi Province, China (recalcitrant organic compounds)	This study
*Microbacterium hydrocarbonoxydans* TY3211y	Jinmen City, Hubei Province, China (petroleum products)	This study
*Microbacterium hydrocarbonoxydans* TY3517	Jinmen City, Hubei Province, China (petroleum products)	This study
*Paenibacillus polymyxa* KM2501	Hukou County, Jiangxi Province, China (recalcitrant organic compounds)	This study
*Paenibacillus polymyxa* KM4410	Qidong City (near Dongyuan), Jiangsu Province, China (excessive salinization)	This study
*Pseudomonas* sp. TY1205	Daye County, Hubei Province, China (heavy metals)	This study
*Pseudomonas* sp. TY1210	Daye County, Hubei Province, China (heavy metals)	This study
*Pseudomonas* sp. KM3113	Jinmen City, Hubei Province, China (petroleum products)	This study
*Pseudomonas* sp. KY5404	Qidong City (near Yinyang), Jiangsu Province, China (excessive salinization)	This study
*Pseudomonas chlororaphis* KY5406	Qidong City (near Yinyang), Jiangsu Province, China (excessive salinization)	This study
*Pseudomonas* sp. TY3101	Jinmen City, Hubei Province, China (petroleum products)	This study
*Pseudomonas* sp. TY3116w	Jinmen City, Hubei Province, China (petroleum products)	This study
*Pseudomonas* sp. KY3201	Jinmen City, Hubei Province, China (petroleum products)	This study
*Pseudomonas* sp. TY5511	Qidong City (near Yinyang), Jiangsu Province, China (excessive salinization)	This study
*Pseudomonas* sp. KM2404	Hukou County, Jiangxi Province, China (recalcitrant organic compounds)	This study
*Pseudomonas protegens* TM1109	Daye County, Hubei Province, China (heavy metals)	This study
*Pseudomonas protegens* TY1502	Daye County, Hubei Province, China (heavy metals)	This study
*Pseudomonas protegens* TY1508	Daye County, Hubei Province, China (heavy metals)	This study
*Pseudomonas protegens* KY4410	Qidong City (near Dongyuan), Jiangsu Province, China (excessive salinization)	This study
*Serratia plymuthica* KM3407	Jinmen City, Hubei Province, China (petroleum products)	This study
*Serratia plymuthica* TM4307-1	Qidong City (near Dongyuan), Jiangsu Province, China (excessive salinization)	This study
*P. brassicacearum* Q8r1-96	Quincy, WA, USA (wheat rhizosphere)	Weller *et al*., 2007; Loper *et al*., 2012
*P. chlororaphis* 30–84	Glen Elder, KS, USA (wheat rhizosphere)	Pierson and Thomashow, 1992
*P. protegens* Pf-5	Texas (cotton rhizosphere)	Paulsen *et al*., 2005
*P. chlororaphis* TX-1	East Lansing, MI (thatch and soil of Kentucky bluegrass)	Powell *et al*., 2000

a Strains number prefixes: KM, isolated from the wheat rhizosphere on ^1^/_3_ × KMB agar; TM, isolated from the wheat rhizosphere on ^1^/_10_ × TSA; KY, isolated from the rapeseed rhizosphere on ^1^/_3_ × KMB agar; TY, isolated from the rapeseed rhizosphere on ^1^/_10_ × TSA.

**Table 3:** Biocontrol related genes and traits of Chinese strains and *in vitro* inhibition of soilborne pathogens^a^

Strain	Antibiotic biosynthesis genes				Extracellular metabolites			Inhibition of pathogen		
	*phlD*	*phzF*	*prnD*	*pltC*	Cel	Sid	Pro	Ggt ARS-A1	Rs AG-8	Ss
*Bacillus* sp. TY5107	**−**	**−**	**−**	**−**	**−**	**+**	**−**	**−**	**−**	**++**
*D. tsuruhatensis* TM3205	**−**	**−**	**−**	**−**	**+**	**+**	**−**	**++**	**−**	**−**
*L. capsici* TM5405	**−**	**−**	**−**	**−**	**−**	**+**	**+**	**+++**	**++**	**−**
*L. enzymogenes* TM2502	**−**	**−**	**−**	**−**	**−**	**+**	**+**	**++**	**++**	**−**
*M. hydrocarbonoxydans* TY3211y	**−**	**−**	**−**	**−**	**+**	**−**	**+**	**−**	**−**	**+**
*M. hydrocarbonoxydans* TY3517	**−**	**−**	**−**	**−**	**−**	**+**	**+**	**−**	**−**	**+**
*Pa. polymyxa* KM2501	**−**	**−**	**−**	**−**	**+**	**+**	**+**	**++**	**++**	**++**
*Pa. polymyxa* KM4410	**−**	**−**	**−**	**−**	**+**	**+**	**+**	**++**	**++**	**++**
*Pseudomonas* sp. TY1205	**−**	**−**	**−**	**−**	**+**	**+**	**−**	**++**	**+**	**+**
*Pseudomonas* sp. TY1210	**−**	**−**	**−**	**−**	**+**	**+**	**−**	**++**	**−**	**++**
*Pseudomonas* sp. KM3113	**−**	**−**	**−**	**−**	**+**	**+**	**+**	**++**	**−**	**+**
*Pseudomonas* sp. KY5404	**−**	**−**	**−**	**−**	**+**	**+**	**−**	**−**	**−**	**+**
*P. chlororaphis* KY5406	**−**	***+***	**+**	**−**	**+**	**+**	**−**	**+**	**+**	**+**
*Pseudomonas* sp. TY3101	**−**	**−**	**−**	**−**	**+**	**+**	**−**	**−**	**++**	**+**
*Pseudomonas* sp. TY3116w	**−**	**−**	**−**	**−**	**+**	**+**	**+**	**−**	**++**	**−**
*Pseudomonas* sp. KY3201	**−**	**−**	**−**	**−**	**+**	**+**	**−**	**−**	**−**	**++**
*Pseudomonas* sp. TY5511	**−**	**−**	**−**	**−**	**+**	**++**	**+**	**−**	**−**	**+**
*Pseudomonas* sp. KM2404	**−**	**−**	**−**	**−**	**+**	**+**	**−**	**−**	**+**	**++**
*P. protegens* TM1109	**+**	**−**	**+**	**+**	**+**	**+**	**−**	**++**	**−**	**+**
*P. protegens* TY1502	**+**	**−**	**+**	**+**	**+**	**+**	**−**	**++**	**−**	**+**
*P. protegens* TY1508	**+**	**−**	**+**	**+**	**+**	**+**	**−**	**++**	**−**	**++**
*P. protegens* KY4410	**+**	**−**	**+**	**+**	**+**	**+**	**−**	**++**	**−**	**++**
*S. plymuthica* KM3407	**−**	**−**	**+**	**−**	**+**	**+**	**−**	**+++**	**+++**	**++**
*S. plymuthica* TM4307-1	**−**	**−**	**+**	**−**	**+**	**+**	**+**	**+++**	**+++**	**++**

a Presence (+) or absence (**−**) of genes required for the biosynthesis of 2,4- DAPG (*phlD*), phenazine (*phzF*), pyrrolnitrin (*prnD*) and pyoluteorin (*pltC*). Extracellular metabolites produced: cellulase (Cel), protease (Pro) and siderophores (Sid).

Isolates were tested *in vitro* for ability to inhibit the growth *of R. solani* AG-8 (Rs AG-8); *G. graminis var. tritici* ARS-A1 (Ggt ARS-A1); and *S. sclerotiorum* (Ss). Inhibition was scored as follows: **−**, no inhibition; + , inhibition zone of < 5 mm; ++, inhibition zone of 5 to 10 mm; +++; and inhibition zone of > 10 mm.

Results of traditional microbiological tests and analysis of partial 16S rDNA gene sequences (Table 2) revealed that five of the 24 strains were Gram-positive and closely related to *Bacillus, Paenibacillus polymyxa* and *Microbacterium hydrocarbonoxydans*. The rest of the strains represented two different clades of Gram-negative bacteria and were closely related to several species within the *Pseudomonas fluorescens* and *Pseudomonas putida* groups, and to *Lysobacter capsici, Lysobacter enzymogenes*, *Serratia plymuthica* and *Delftia tsuruhatensis*. We subjected strains of the *P. fluorescens* group to the multilocus typing scheme of Mulet and colleagues (2009; 2010), which has proven to be useful for establishing phylogenetic relationships among pseudomonads and is based on sequences of 16S rDNA, *gyrB* and *rpoD* (Mulet *et al*., 2009; 2010). The results of molecular typing identified strains TY1502, TY1508, KY4410 and TM1109 as *P. protegens* and strain KY5406 as *P. chlororaphis* (Fig. S1). Strains TY1205, TY1210, KM3113 and KY5404 were identified as members of the *P. corrugata* subgroup and were closely related but not identical to the well-studied biocontrol species *P. brassicacearum* (Fig. S1).

All 24 strains produced one or more of the extracellular metabolites cellulase, protease and siderophores. Genes for the biosynthesis of the antibiotics 2,4-DAPG (*phlD*), phenazines (*phzF*), pyrrolnitrin (*prnD*) and/or pyoluteorin (*pltC*) were detected in strains of *P. protegens*, *P. chlororaphis* and/or *S. plymuthica* (Table 3) and the antibiotics themselves were detected in culture extracts analysed by thin-layer chromatography. These antibiotics are well known to function as major mechanisms of biocontrol of soilborne pathogens.

The amplified fragments of *phlD*, *phzF*, *prnD* and *pltC* from these strains were sequenced and used in phylogenetic analyses together with corresponding alleles previously deposited in GenBank. Phylograms inferred from *phlD* and *pltC* sequences revealed that both of these genes from strains TM1109, TY1502, TY1508 and KY4410 were closely related to their homologues from *P. protegens* (Figs S2 and S3). Strains TM1109, TY1502, TY1508 and KY4410 also carried a *P. protegens-*like *prnD* allele and therefore had a combination of antibiotic biosynthesis pathways similar to those of the model biocontrol agents *P. protegens* strains Pf-5 and CHA0 (Fig. S4). The second *prnD-*positive *Pseudomonas* strain, KY5406, also carried *phzF* (Table 3), a gene required for the production of phenazine antibiotics and highly conserved in all known phenazine producers. The *prnD* and *phzF* genes of KY5406 clustered with homologues from *P. chlororaphis* (Figs S4 and S5). Strain KY5406 also contained *phzO* (data not shown), a gene required for the biosynthesis of 2-hydroxylated phenazine compounds, indicating this strain is subsp. *aureofaciens*. Finally, two more *prnD-*positive strains, KM3407 and TM4307-1 (Table 3), carried *prnD* alleles similar to those present in pyrrolnitrin-producing strains of *Serratia* spp. (Fig. S4) and produced Prn *in vitro* (data not shown). In total, correlation of the antibiotic gene profiling with the taxonomic placement of the strains by partial 16S rRNA gene sequence analysis identified the antibiotic-producing strains as members of *P. protegens*, *P. chlororaphis* subsp. *aureofaciens* and *S. plymuthica*.

1-aminocyclopropane-1-carboxylic acid (ACC) deaminase and IAA production by PGPR are important determinants in plant–bacterial interactions, and secretion of these compounds can induce plants to produce longer and more vigorous roots (Penrose and Glick, 2003). *Delftia tsuruhatensis* TM3205 and *Pseudomonas* strains KM3113, KY5404, TY1210, TY1205, KY3201 and TY3101 produced IAA in culture. PCR analysis demonstrated that the aforementioned *Pseudomonas* strains were positive for *acdS*, a gene required for the production of ACC deaminase (Table 4), and the sequences of those strains closely resembled those from other fluorescent pseudomonads (Fig. S6). All *acdS* sequences from the Chinese strains formed a distinct clade within the *Pseudomonas* group and clustered tightly with *acdS* from *Pseudomonas* sp. 2–3 (GenBank Accession No. EU520401). All *acdS*-positive strains grew in defined medium supplemented with ACC as a sole nitrogen source.

**Table 4:** Growth promotion of canola roots by rhizobacteria applied as seed treatments in growth pouch experiments^a^

Treatment	*acdS*	IAA	Exp 1 Root length	Increase (%)	Exp 2 Root length	Increase (%)	Exp 3 Root length	Increase (%)
*Bacillus* sp. TY5107	**−**	**−**	6.8 ± 0.5 BE	20.2	4.2 ± 0.4 GH	**−**18.5	4.8 ± 0.3 I	**−**29.4
*D. tsuruhatensis* TM3205	**−**	+	8.1 ± 0.4 AD	43.3	5.7 ± 0.4 DE	10.8	9.7 ± 0.3 A	42.7
*L. capsici* TM5405	**−**	**−**	7.0 ± 0.3 BE	22.5	NT		5.8 ± 0.4 H	**−**13.6
*L. enzymogenes* TM2502	**−**	**−**	NT		4.9 ± 0.4 EG	**−**4.5	5.9 ± 0.3 H	**−**13.5
*M. hydrocarbonoxydans* TY3211y	**−**	**−**	7.9 ± 0.3 AD	38.8	5.5 ± 0.4 DF	7.1	7.7 ± 0.4 FG	13.9
*M. hydrocarbonoxydans* TY3517	**−**	**−**	5.8 ± 0.2 DE	2	5.4 ± 0.3 DF	5.2	6.5 ± 0.3 H	**−**3.5
*Pa. polymyxa* KM2501	**−**	**−**	7.4 ± 0.3 AE	30.9	4.6 ± 0.4 FG	**−**11.7	NT	
*Pa. polymyxa* KM4410	**−**	**−**	3.1 ± 0.3 E	**−**45.8	4.9 ± 0.4 EG	**−**5.2	NT	
*Pseudomonas* sp. KM3113	+	+	8.1 ± 0.4 AD	42.7	7.0 ± 0.4 AB	35.6	9.4 ± 0.4 AB	38.4
*Pseudomonas* sp. KY5404	+	+	8.5 ± 0.5 AC	50	7.9 ± 0.4 A	53.4	8.2 ± 0.3 DF	21.6
*Pseudomonas* sp. TY1210	+	+	9.6 ± 0.4 A	68.3	7.0 ± 0.3 AB	35.7	8.4 ± 0.4 CDF	23.5
*Pseudomonas* sp. TY1205	+	+	9.4 ± 0.5 A	64.8	7.0 ± 0.3 AB	35.4	9.3 ± 0.4 AC	37.8
*P. chlororaphis* KY5406	**−**	**−**	6.0 ± 0.4 CE	5.9	4.4 ± 0.4 GH	**−**15.6	5.9 ± 0.4 H	**−**13
*Pseudomonas* sp. KY3201	+	+	9.7 ± 0.3 A	71.4	5.9 ± 0.3 CD	14.8	7.9 ± 0.4 EF	17.5
*Pseudomonas* sp. TY3101	+	+	8.4 ± 0.5 AB	48.8	5.5 ± 0.4 DF	6.9	9.7 ± 0.4 A	43
*Pseudomonas* sp. TY3116w	**−**	**−**	8.1 ± 0.2 AD	42.9	5.8 ± 0.3 DE	12.2	7.6 ± 0.4 FG	12.7
*Pseudomonas* sp. TY5511	**−**	**−**	8.3 ± 0.3 AD	45.9	5.7 ± 0.3 DE	10.6	8.8 ± 0.3 AE	29.9
*Pseudomonas* sp. KM2404	**−**	**−**	8.4 ± 0.3 AC	47.9	7.0 ± 0.4 AB	36	9.7 ± 0.4 DF	42.7
*P. protegens* KY4410	**−**	**−**	7.9 ± 0.3 AD	39.9	7.6 ± 0.3 AB	47.2	8.8 ± 0.4 AE	29.8
*P. protegens* TM1109	**−**	**−**	9.1 ± 0.4 AB	60.6	7.6 ± 0.3 AB	47.5	8.5 ± 0.3 BF	25.7
*P. protegens* TY1502	**−**	**−**	9.1 ± 0.4 AB	60.6	6.8 ± 0.3 BC	31.7	8.6 ± 0.4 BF	27.2
*P. protegens* TY1508	**−**	**−**	8.7 ± 0.5 AB	53.6	7.4 ± 0.3 AB	42.2	9.0 ± 0.4 AD	33.2
Ck	**−**	**−**	5.7 ± 0.5 CE		5.2 ± 0.4 DG		6.8 ± 0.3 GH	

a Surface-sterilized canola seeds were soaked for 1 h in each bacterial suspension. Control (CK), non-treated seeds were soaked in sterile water.

Presence (+) or absence (**−**) of the *acdS* gene. Bacterial cultures were positive (+) or negative (**−**) for the presence of IAA.

% increase = [(root length of a treatment- root length of the control)/ root length of the control] × 100%. Means in the same column followed by the same letter are not significantly different at *P* = 0.05 according to Fisher's protected least significant difference test (LSD) or Kruskal–Wallis all pairwise comparison test.

Exp, experiment; NT, not tested.

### Biocontrol and growth-promoting activity of the rhizobacterial strains

A subset of strains with the highest antagonistic activity and representative of the dominant morphotypes was selected to determine their biocontrol activity against take-all and Rhizoctonia root rot of wheat under greenhouse conditions in sterile, pasteurized and raw soils from Wuhan, China and Quincy, Washington. Most of the isolates provided some levels of reduction in root disease as compared with the pathogen-inoculated controls (Tables 5–7). However, strains identified as *L. capsici*, *L. enzymogenes*, *P. protegens* and *P. chlororaphis* provided the best and most consistent disease suppression across experiments and pathogens. *Pseudomonas protegens* TM1109 was especially effective in biocontrol of both take-all and Rhizoctonia root rot. These results indicate that indigenous rhizobacteria from the contaminated soil have the capacity to provide protection of plants against soilborne fungal pathogens. Surprisingly, application of *S. plymuthica* TM4307-1, which highly antagonized the pathogens *in vitro* (Table 3), resulted in significantly higher levels of Rhizoctonia root rot (Table 6). We speculate that TM4307-1 produces yet unidentified phytotoxic metabolite(s) since treatment of both wheat and canola with this bacterium resulted in significant stunting and bleaching of the seedlings and root damage.

**Table 5:** Suppression of *Rhizoctonia solani* AG-8 in sterile soil in greenhouse pot experiments^a^

Treatment	Disease rating		Shoot length (cm)	
	Exp 1	Exp 2	Exp 1	Exp 2
CK1	0.4 ± 0.1	0.3 ± 0.5	25.3 ± 4.9	36.4 ± 2.7
CK2	5.0 ± 1.6 A	4.6 ± 1.3 AB	20.5 ± 5.3 D	33.3 ± 3.6 BC
*L. capsici* TM5405	3.3 ± 1.6 D	3.1 ± 1.7 C	23.6 ± 4.6 ABC	37.1 ± 3.9 A
*L. enzymogenes* TM2502	3.7 ± 1.7 CD	3.7 ± 1.7 BC	23.1 ± 5.0 ABC	31.7 ± 4.3 C
*Pa. polymyxa* KM2501	4.1 ± 1.8 ABC	3.6 ± 1.5 BC	22.6 ± 4.4 BC	33.9 ± 4.1 B
*Pa. polymyxa* KM4410	3.1 ± 1.7 D	4.6 ± 1.3 AB	24.4 ± 4.7 A	34.8 ± 5.3 B
*P. protegens* TM1109	3.8 ± 1.6 BCD	3.8 ± 1.7 BC	23.7 ± 4.7 AB	31.7 ± 3.4 C

a CK1, control consisting of sterile soil from Wuhan, China not amended with inoculum of *R. solani* and sown to non-treated seeds; CK2, control consisting of soil amended with *R. solani* inoculum and sown to methyl cellulose coated seeds.

Severity of Rhizoctonia root rot was evaluated on a scale of 0–8. Means in the same column followed by the same letter are not significantly different at *P* = 0.05 according to Fisher's protected least significant difference test (LSD) or Kruskal–Wallis all pairwise comparison test.

Exp, experiment.

**Table 6:** Suppression of *Rhizoctonia solani* AG-8 in Quincy virgin soil in a growth chamber tube assays^a^

Treatment	Disease rating			
	Pasteurized soil		Raw soil	
	Exp 1	Exp 2	Exp 3	Exp 4
CK1	0.5 ± 1.5	0.1 ± 0.5	0.3 ± 0.8	0.1 ± 0.3
CK2	4.8 ± 0.8 B	4.9 ± 0.8 B	4.4 ± 0.6 A	4.5 ± 0.7 AB
CK3	4.7 ± 0.9 BC	5.2 ± 0.8 B	4.3 ± 0.6 AB	4.6 ± 0.6 A
*D. tsuruhatensis* TM3205	NE	NE	3.9 ± 0.4 ABC	4.1 ± 0.4 BC
*L. capsici* TM5405	4.3 ± 1.1 BCD	4.8 ± 0.8 B	3.8 ± 0.5 BC	4.1 ± 0.7 C
*L. enzymogenes* TM2502	NE	NE	3.9 ± 0.9 ABC	4.0 ± 0.5 C
*Pa. polymyxa* KM4410	4.4 ± 1.7 BCD	4.9 ± 0.9 B	3.9 ± 0.5 BC	4.0 ± 0.4 C
*Pseudomonas* sp. KM3113	NE	NE	4.2 ± 0.7 AB	4.1 ± 0.6 BC
*P. chlororaphis* KY5406	4.3 ± 0.8 CD	3.9 ± 1.0 C	3.4 ± 1.6 C	3.7 ± 1.8 CD
*Pseudomonas* sp. KM2404	4.4 ± 0.9 BCD	4.8 ± 0.8 B	NE	NE
*P. protegens* KY4410	NE	NE	2.4 ± 1.2 D	3.2 ± 1.1 D
*P. protegens* TM1109	4.2 ± 0.8 D	4.2 ± 0.8 C	2.0 ± 1.2 D	3.1 ± 1.2 D
*S. plymuthica* TM4307-1	6.6 ± 0.9 A	6.3 ± 1.0 A	NE	NE

a CK1, soil not amended with *R. solani* inoculum and sown to non-treated seed; CK2, soil amended with *R. solani* inoculum and sown to methyl cellulose coated seed; and CK3, soil amended with oat-kernel inoculum and sown to non-treated seed.

Severity of Rhizoctonia root rot was evaluated on a scale of 0–8. Experiments 1 and 2 were conducted in Quincy virgin soil that had been pasteurized (60°C, 30 min); experiments 3 and 4 were conducted in raw Quincy virgin soil. Means in the same column followed by the same letter are not significantly different at *P* = 0.05 according to Fisher's protected least significant difference test (LSD) or Kruskal–Wallis all pairwise comparison test.

Exp, experiment; NE, not evaluated.

**Table 7:** Suppression of *G. graminis* var. *tritici* ARS-A1 in Quincy virgin soil in a growth chamber tube assays^a^

	Disease rating			
	Pasteurized soil		Raw soil	
Treatment	Exp 1	Exp 2	Exp 3	Exp 4
CK1	0.5 ± 1.4	0.0 ± 0.3	0.5 ± 1.4	0.0 ± 0.2
CK2	5.2 ± 1.2 AB	6.7 ± 0.8 A	4.5 ± 0.9 ABC	6.1 ± 1.0 AB
CK3	5.3 ± 1.0 AB	6.6 ± 0.8 A	4.7 ± 1.0 AB	6.1 ± 1.0 A
*L. capsici* TM5405	3.8 ± 0.8 D	5.9 ± 1.0 BC	3.3 ± 0.7 E	5.3 ± 1.4 BCD
*Pa. polymyxa* KM4410	5.0 ± 1.3 AB	6.6 ± 0.9 A	4.2 ± 0.9 BC	5.9 ± 1.2 ABC
*P. chlororaphis* KY5406	4.0 ± 1.2 CD	4.8 ± 1.2 D	3.7 ± 1.2 DE	4.4 ± 1.0 E
*Pseudomonas* sp. KM2404	5.2 ± 1.2 AB	6.6 ± 1.0 A	4.0 ± 1.0 CD	5.9 ± 1.0 ABC
*P. protegens* TM1109	3.9 ± 1.1 D	5.4 ± 0.9 CD	3.2 ± 0.8 E	4.6 ± 1.3 DE
*S. plymuthica* TM4307-1	5.8 ± 1.3 A	6.6 ± 0.9 A	5.3 ± 1.6 A	6.1 ± 1.4 AB

a CK1, Quincy virgin soil not amended with inoculum and sown to non-treated seed; CK2, soil amended with *G. graminis* var *tritici* inoculum and sown to methyl cellulose coated seed; and CK3, soil amended with *G. graminis* var *tritici* inoculum and sown to non-treated seed.

Severity of take-all was evaluated on a scale of 0–8. Experiments 1 and 2 were conducted in pasteurized Quincy virgin soil (60°C, 30 min); experiments 3–4 were conducted in raw Quincy virgin soil. Means in the same column followed by the same letter are not significantly different at *P* = 0.05 according to Fisher's protected least significant difference test (LSD) or Kruskal–Wallis all pairwise comparison test.

Exp, experiment.

Twenty-two of the 24 isolates were tested for the ability to promote canola root growth in growth pouch assays. The two *S. plymuthica* strains were eliminated from this assay because they enhanced disease when applied to wheat and caused stunting. Thirteen strains increased root growth significantly (*P* = 0.05) in at least one of the three experiments (Table 4). *Pseudomonas protegens* and *P. brassicacearum-*like strains demonstrated the most consistent capacity to promote root growth and, on average, increased root length in canola by 41% to 42%, respectively, as compared with the non-treated controls. All *P. brassicacearum-*like isolates produced IAA and carried *acdS*. In contrast, strains of *P. protegens* had neither trait. Other tested *Pseudomonas* strains (i.e. KY3201, TY3101, Ty3116w, Ty5511 and KM2402) increased root length consistently but not always significantly, whereas strains of *Bacillus*, *Paenibacillus* and *Lysobacter* had no growth promotion activity. These results indicate that rhizobacteria from the contaminated soil have the capacity to directly enhance plant growth.

### Tolerance to abiotic stressors

Based on the results from the biocontrol and growth promotion assays, 10 of the 24 strains were selected and tested for tolerance to two heavy metals and high levels of salt by growing them in LB broth supplemented with CdCl_2_, CuCl_2_ and NaCl. All 10 strains tolerated high levels of salt and grew well in up to 5–7% NaCl. Similarly, these 10 strains grew in the presence of heavy metals and demonstrated minimal inhibitory concentrations (MICs) to Cu^2+^ of > 4 mM and Cd^2+^ of > 1 mM. Overall, *P. protegens* TM1109 was the most tolerant to these stressors (Table 8).

**Table 8:** Minimum inhibitory concentrations (MICs) of heavy metals and salt for selected strains^a^

Isolate	Type of soil contamination strain from	Cd^2+^ (mM)	Cu^2+^ (mM)	NaCl (%)
*D. tsuruhatensis* TM3205	Petroleum products	1.3	4.4	7
*Pseudomonas* sp. KM3113	Petroleum products	1	4.4	6
*Pseudomonas* sp. TY1205	Heavy metals	1	4.8	5.5
*Pseudomonas* sp. KY5404	Excessive salinization	1	5	5.5
*Pseudomonas* sp. TY1210	Heavy metals	1	4	6
*P. chlororaphis* KY5406	Excessive salinization	1.5	5	5.5
*Pseudomonas* sp. TY3101	Petroleum products	1	4	5.5
*Pseudomonas* sp. KY3201	Petroleum products	1	4	7.5
*P. protegens* TM1109	Heavy metals	1.5	5	7
*P. protegens* KY4410	Excessive salinization	1.5	4.8	7

a MICs were determined in Luria–Bertani (LB) broth amended with CuCl_2_ to give a range of concentrations from 3 to 6 mM; CdCl_2_ to give a range of concentrations from 0.4 to 2.0 mM; and NaCl to give concentrations ranging from 0% to 8%.

## Discussion

Plants have evolved along with a rhizosphere microbiome that contributes to the growth and health of the plant (Pieterse *et al*., 2014). Beneficial microbes that directly promote growth or defend roots are recruited by the roots from the bulk soil, making soil type an important determinant of the composition of the rhizosphere microbiome. However, the plant also strongly modulates the shape of the microbial community because both the quality and quantity of root exudates are regulated by the plant genotype (Bais *et al*., 2006; Hartmann *et al*., 2009). Our results support the hypothesis that certain groups of PGPR and biocontrol and growth-promoting genes are cosmopolitan and conserved in soil microbiomes worldwide. Driven by rhizodeposition, the plant enriches and supports PGPR best adapted to colonize the roots, antagonize pathogens, stimulate growth and/or initiate pattern-triggered immunity.

We tested our hypothesis by characterizing culturable PGPR in the rhizospheres of wheat and rapeseed grown in soils from polluted fields, and assessed their growth-promoting and biocontrol abilities. After the most abundant bacterial morphotypes were isolated, the only other criterion we used for selecting the rhizobacteria was the ability to inhibit *G. graminis* var. *tritici*, *R. solani* or *S. sclerotiorum*. This was done because *in vitro* inhibition of plant pathogens is a characteristic commonly found in many PGPR strains (Haas and Défago, 2005) even though antibiosis does not predict biocontrol or growth-promoting activity. The 24 strains described here were shown to be most closely related to species known to have members with PGPR activity. Common among these strains were genes and traits that are well known to contribute to biocontrol and growth-promoting activity (Haas and Défago, 2005; Glick *et al*., 2007; Loper *et al*., 2007; Lugtenberg and Kamilova, 2009). For example, *phzF*, detected in *P. chlororaphis* subsp. *aureofaciens* KY5406, is a key gene in the biosynthesis of phenazine-1-carboxylic acid (PCA) and serves as a marker for the capacity of PGPR to produce one or more phenazines, a large class of broad-spectrum antibiotics produced by a variety of bacteria (Mavrodi *et al*., 2006; 2010). Yang and colleagues (2011) showed that *phzF*-containing and PCA-producing *Pseudomonas* spp. colonized wheat grown in Hebei and Jiangsu provinces, China, and that these strains suppressed take-all. In the Pacific Northwest of the United States, PCA-producing pseudomonads are abundant on the roots of dryland wheat and suppress take-all and Rhizoctonia root rot (Thomashow and Weller, 1988; Mavrodi *et al*., 2012). Phenazine-producing PGPR also contributed to the natural suppressiveness of soils in the Châteaurenard region of France to Fusarium wilt (Mazurier *et al*., 2009). *phlD* is a key gene in the biosynthesis of the broad-spectrum antibiotic 2,4-DAPG in *Pseudomonas* spp. and 2,4-DAPG producers are responsible for the natural suppression of take-all of wheat in the United States and black root rot of tobacco in soils of the Morens region of Switzerland (Weller *et al*., 2007). 2,4-DAPG also induces systemic resistance in plants to pathogens (Weller *et al*., 2012). *prnD* and *pltC* are genes that encode for the biosynthesis of pyrrolnitrin and pyoluteorin, antibiotics that are highly active against *Rhizoctonia* and *Pythium* species respectively (Loper *et al*., 2007). The gene *acdS* encodes ACC deaminase, which promotes plant growth and development by decreasing ethylene levels in the plant. ACC deaminase-producing PGPR convert the ethylene precursor ACC to α-ketobutyrate and ammonia and can relieve stress resulting from pathogens, polyaromatic hydrocarbons, heavy metals, salt and drought (Glick *et al*., 2007).

To validate the biocontrol activity of our strains, we used the Rhizoctonia root rot- and take-all-wheat pathosystems. Strains of *P. protegens*, *P. chlororaphis* subsp. *aureofaciens* and *L. capsici* provided the most consistent disease suppression and members of all three species are biocontrol agents of a broad range of diseases. For example, *P. protegens* Pf-5 produces a wide arsenal of antibiotics and toxins and approximately 6% of its genome is devoted to the production of secondary metabolites (Paulsen *et al*., 2005; Loper *et al*., 2007). Strain CHA0, isolated from a tobacco black root rot suppressive soil, is another notable *P. protegens* with exceptional biocontrol activity (Ramette *et al*., 2011). Our four *P*. *protegens* strains, TM1109, TY1502, TY1508 and KY4410, contained the three antibiotic biosynthesis genes, *phlD*, *prnD* and *pltC*, and produced the antibiotics that are characteristic of Pf-5 and CHA0.

*Pseudomonas chlororaphis* subsp. *aureofaciens* contains *phzO* (Mavrodi *et al*., 2010), and besides PCA, it also produces hydroxyphenazines, which have characteristic orange and red colours and differ from PCA in antibiotic activity (Mavrodi *et al*., 2010). Notable examples of *P. chlororaphis* subsp. *aureofaciens* with biocontrol activity include strains 30–84 (Pierson and Thomashow, 1992) and TX-1 (Powell *et al*., 2000). In *P. chlororaphis*, phenazine production also contributes to rhizosphere competence (Mavrodi *et al*., 2006). *Lysobacter* sp. strains also have broad antifungal and antibacterial activities. For example, Park and colleagues (2008) reported that *L. capsici* YC5194^T^ inhibited *Pythium ultimum*, *Colletotrichum gloeosporioides*, *Fusarium oxysporum*, *Botrytis cinerea*, *R. solani*, *Botryosphaeria dothidea* and *Bacillus subtilis*. *Lysobacter* sp. SB-K88 (formerly *Stenotrophomonas*) (Nakayama *et al*., 1999) produced the antibiotics xanthobaccins A, B and C, and xanthobaccin A was involved in the suppression of Pythium damping-off of sugar beet (Islam *et al*., 2005).

To test our strains for the ability to directly promote plant growth, we used the canola root elongation assay, a model system commonly employed to demonstrate direct growth-promoting activity by PGPR (Patten and Glick, 2002). The most consistent and effective growth promoters were strains of *Delftia* and *Pseudomonas* that produced IAA and, in the case of *P. fluorescens-* and *P. brassicacearum*-like strains, harboured *acdS*. Of these species, only *P. fluorescens* and *P. brassicacearum* have been reported to have direct growth-promoting ability (Belimov *et al*., 2007; Glick *et al*., 2007). Members of these two species also are biocontrol agents (Loper *et al*., 2012). *Pseudomonas brassicacearum* has commonly been isolated from *Arabidopsis thaliana*, *Brassica napus* (Achouak *et al*., 2000) and wheat (Ross *et al*., 2000). *Pseudomonas brassicacearum* strain Am3, with ACC deaminase activity, promoted root elongation of Indian mustard and increased root and shoot biomass of rapeseed and pea (Belimov *et al*., 2001; Safronova *et al*., 2006). A mutation in *acdS* resulted in a loss of growth-promoting activity in *P. brassicacearum* (Belimov *et al*., 2007).

We tested 10 strains for tolerance to heavy metals and high levels of salt by growing them in broth supplemented with CdCl_2_, CuCl_2_ and NaCl. Unfortunately, there are no records of how long the sampled fields have been polluted but discussions with local inhabitants indicated that some fields may have been in that condition for decades. Not surprising, all of the PGPR strains tested tolerated high levels of salt and demonstrated MICs to Cu^2+^ and Cd^2+^ on par or higher than those previously reported for strains adapted to survival in the presence of acute levels of heavy metal contamination of soil (Thomas *et al*., 2008; Altimira *et al*., 2012). Thus, our results confirm that PGPR species have adapted not only to a wide variety of cropping systems and crops but also to adverse environmental conditions. Of particular note are our strains of *P. protegens*, which appeared to be especially well adapted to a wide variety of pollutants. Perhaps not surprisingly, analysis of the genome of *P. protegens* Pf-5 identified a considerable collection of efflux systems that provide protection against a range of toxic metabolites (Loper *et al*., 2007). Evidence is accumulating that strains of *P. protegens* form the cornerstones of communities of microbial defenders of roots in rhizosphere microbiomes globally.

In conclusion, Chinese farmers often must utilize all arable land, even polluted fields, because of the challenges of feeding a huge population. We inventoried PGPR in contaminated soils from sites representative of polluted fields supporting agricultural production. These sites were especially interesting because their microbiology previously had not been studied. Our findings demonstrate that well-described PGPR species and biocontrol and growth-promoting genes are common in the rhizosphere microbiome of plants grown in polluted Chinese soils. PGPR from such soils may be the best source of strains for commercial development given that in China nearly 50 million acres of arable land suffer from some type of soil pollution (Zhou, 2013). With the world population expected to be 9–11 billion by 2050, the challenge is to identify crop cultivars and cropping systems that make fuller use of indigenous and introduced PGPR for pest protection and growth promotion since the amount of arable land and water available for food production will likely continue to decrease.

## Experimental procedures

### Bacterial strains, soilborne pathogens and growth media

Bacterial strains used in this study are listed in Table 2. *Pseudomonas* isolates were grown in KMB or one-third-strength KMB (^1^/_3_ × KMB) agar or broth at 28°C as described by Mavrodi and colleagues (2012). Other strains were grown in TSA or tryptic soy broth (TSB) or these media at one-tenth strength (^1^/_10_ × TSB or TSA) (Mavrodi *et al*., 2012) at 28°C. When needed to inhibit fungi, these media were supplemented with cycloheximide (100 μg ml^−1^). All strains were stored in 60% glycerol at −80°C. The fungal wheat root pathogens *Gaeumannomyces graminis* (Sacc.) von Arx and Olivier var. *tritici* Walker isolate ARS-A1 (Yang *et al*., 2011) and *R. solani* Kühn [teleomorph *Thanatephorus cucumeris* (Frank) Donk] AG-8 isolate C-1 (Huang *et al*., 2004), and the rapeseed/canola pathogen *S. sclerotiorum* (Lib.) de Bary isolate MGSCF180002 (from rapeseed) were grown at room temperature on one-fifth-strength homemade potato dextrose agar (^1^/_5_ × PDA) (Yang *et al*., 2011). *In vitro* inhibition assays of pathogens by PGPR were conducted on full-strength PDA.

### Sample collection

Soil was sampled from five polluted sites in China: farmland contaminated with heavy metals in Daye County, Hubei Province; a riverside field at a recalcitrant organic sewage outlet in Gold Sand Bay Industrial Park, Hukou County, Jiangxi Province; farmland beside a petroleum products sewage outlet in Jinmen City, Hubei Province; and saline-alkali fields on two separate seaside farms in Qidong City (near Dongyuan and Yinyang respectively), Jiangsu Province. At each site, plants were dug to a depth of 30 cm from five random locations. Two kilograms of soil surrounding the roots of plants from each location was placed in plastic bags, transported to the laboratory and stored at 4°C until processed as described below.

### Wheat and rapeseed greenhouse cycling assays in contaminated soils

Soil from each location was sieved, put into three square pots (6.5 cm high × 7 cm wide) and 10 ml of a metalaxyl (Syngenta, Wilmington, DE, USA) solution (75 mg l^−1^) was added to prevent damping-off disease caused by indigenous *Pythium* spp. Each pot served as a replicate. Six surface-sterilized seeds of wheat (cv. Zhengmai 9023) or rapeseed (cv. Xiangyou 571) were pre-germinated for 48 h, sown in each pot and then covered with a 1.5 cm-thick layer of sterile vermiculite. Each pot was then given 30 ml of water. The pots were covered with a plastic bag for 4 days and incubated in a greenhouse (15–18°C; dark/light cycle, 12 h). Each pot received 30 ml of water two times weekly and diluted (1:3, vol/vol) Hoagland's solution once weekly. After 3 weeks, the seedlings were removed from the pots, roots were excised and soil and roots from all of the pots of the same treatment were mixed and added back to the pots and sown as before with wheat or rapeseed to begin a second growth cycle. At the end of the third growth cycle, roots from two randomly chosen plants from each pot were selected for enumeration of populations of total culturable aerobic, heterotrophic rhizobacteria (total bacteria) by the end-point dilution assay as described below.

### Enumeration and isolation of rhizosphere bacteria

Roots with adhering rhizosphere soil of two plants from the same pot were excised and placed in a 50 ml screw-cap tube with 10 ml of sterile distilled water, vortexed (1 min) and sonicated in an ultrasonic cleaner (1 min) to dislodge rhizobacteria (Mavrodi *et al*., 2012). The dilution endpoint assay with ^1^/_10_ × TSB plus cycloheximide was used to determine the population density of total bacteria as described by Mavrodi and colleagues (2012). The 96-well microplates were incubated at room temperature in the dark, and after 72 h, each well was scored for growth. At the end of the 3rd growth cycle, rhizobacteria were isolated by dilution plating of the root washings onto ^1^/_3_ × KMB agar and ^1^/_10_ × TSA, each with cycloheximide. Plates were incubated at room temperature for 2 to 3 days or until colonies were visible. Colonies of different morphotypes were re-streaked onto ^1^/_3_ × KMB and ^1^/_10_ × TSA plates and stored in 60% glycerol at −80°C.

### Pathogen inhibition *in vitro*

Inhibition of soilborne pathogens by rhizobacteria was tested on PDA by two approaches. In the first, aliquots (20 μl) from overnight broth cultures were introduced into a hole in the agar cut with a 5 mm cork borer, 1 cm from the edge of the Petri dish. A 5 mm plug from a culture of *R. solani* AG-8 or *S. sclerotiorum* grown on ^1^/_5_ × PDA was placed in the centre of the plate. Plates were incubated at 28°C and scored after 4 days by measuring the distance between the edges of the bacteria and the fungal mycelium. Four isolates were tested on each plate and each isolate was tested three times. In a second approach, aliquots (2 μl) from overnight cultures were spotted twice, 1 cm from the edge of a plate of PDA, and a plug of *R. solani* AG-8 was placed in the centre. For tests against *G. graminis* var. *tritici*, the plug of the fungus was placed in the centre of the plate 24 h before the bacteria were spotted. The zone of inhibition was measured 5 days later. Each isolate was tested four times.

### Morphological and physiological characteristics of isolates

Selected isolates were characterized based on morphological and biochemical tests as described by Zhao and He (2012). Morphological characteristics included colony morphology, pigmentation, cell shape and Gram stain reaction. Biochemical and physiological characterization included tests for starch hydrolysis, gelatin liquefaction, catalase production, pectin decomposition, indole production and production of phenazine-1-carboxylic acid, 2,4-DAPG, pyrrolnitrin and pyoluteorin. Cellulase activity was determined on carboxymethylcellulose (CMC) medium (per 1 l: peptone, 10 g; yeast extract, 10 g; sodium CMC, 10 g; NaCl, 5 g; KH_2_PO_4,_ 1 g; agar, 15 g; pH 7.0). A 10 μl aliquot of an overnight bacterial culture was spotted on sterile filter paper (5 mm diameter, 0.5 mm thick) on the surface of CMC agar. To visualize the zone of hydrolysis, plates were incubated at 28°C for 48 h, flooded with an aqueous solution of 0.1% Congo red for 1 h and washed with 1 M NaCl (Ghose, 1987). Protease activity, demonstrated by casein degradation, was indicated by the appearances of zones of clearing around colonies grown on milk agar (per 1 l: dry skim milk, 100 g; agar, 20 g) after incubation at 28°C for 3 days. Siderophores were detected on CAS (chrome azurols) agar plates (Shin *et al*., 2011). Antibiotics (phenazine-1-carboxylic acid, pyrrolnitrin, pyoluteorin and DAPG) were isolated from bacterial cultures grown for 5 days on KMB agar by a modification of procedures described by Kraus and Loper (1992). Briefly, cells and spent agar (*c*. 15 ml) were macerated with a mortar and pestle in an equal volume of acetone. The acetone was evaporated in a hood overnight and the residual aqueous phase was extracted twice with equal volumes of ethyl acetate. The ethyl acetate was recovered, dried and the residue was dissolved in 50 μl of methanol. Samples (5 μl) of the extracts, standards of the four antibiotics, and positive control extracts from strains *P. protegens* Pf-5 and *P. brassicacearum* Q8r1-96 were spotted on silica gel (GHLF) thin layer chromatography plates (Analtech, Newark, DE, USA). The plates were developed with chloroform/acetone (9:1) and observed under UV light (254 nm).

### Amplification and sequencing of 16S rDNA, antibiotic biosynthesis and ACC deaminase genes

Genomic DNA was extracted from strains using a QIAamp DNA Mini Kit (Qiagen, Valencia, CA, USA) and quantified by fluorometry using a DNA quantitation kit (Bio-Rad, Hercules, CA, USA). Genes encoding 16S rDNA, subunit B of DNA gyrase, sigma 70 factor of RNA polymerase and key antibiotic biosynthesis enzymes (i.e. *phzF*, *phlD*, *prnD* and *pltC*) were amplified by using published oligonucleotide primer sets (McSpadden Gardener *et al*., 2001; De Souza and Raaijmakers, 2003; Mulet *et al*., 2009; Mavrodi *et al*., 2010) and cycling conditions (Table 9). The ACC deaminase gene (*acdS*) was targeted with primers F1936f-tail and F1938r-tail. These primers were derived from primers F1936 and F1938 (Blaha *et al*., 2006) by adding to their 5′-ends sequences GCTCCTACTCTGTCACCTATC and CTGTCGCTCTGGCTGTC, respectively, for direct sequencing of *acdS* amplicons with primers Tail1 and Tail2 (Table 9). Amplification conditions for *acdS* included initial denaturation at 94°C for 1 min, followed by 10 cycles of 94°C for 30 s, 53°C for 20 s and 72°C for 45 s, followed by 25 more cycles with primer annealing temperature of 60°C. All amplifications were performed with a PTC-200 gradient thermal cycler (Bio-Rad) using GoTaq DNA polymerase (Promega, Madison, WI, USA). The amplifications were carried out in 25 μl reactions that contained 100 ng of DNA; 1 × GoTaq Flexi buffer, 200 μM dNTPs, 1.5 mM MgCl_2_, 20 pmol of each primer pair and 1.2 U of GoTaq Flexi DNA polymerase (Promega). Positive controls included DNA from *P. chlororaphis* subsp. *aureofaciens* TX-1 (*phzF*), *P. brassicacearum* Q8r1-96 (*phlD*, *acdS*) and *P. protegens* Pf-5 (*phlD*, *prnD*, *pltC*). Amplification products were cleaned with QIAquick PCR purification spin columns (Qiagen) and sequenced directly with a BigDye Terminator v. 3.1 cycle sequencing kit (Applied Biosystems, Foster City, CA, USA) at ELIM Biopharmaceuticals (Hayward, CA, USA).

**Table 9:** Target genes and PCR and primers used in this study

Gene	Primer	Sequence (5′-3′)	Annealing temp (°C)	Putative gene function	Amplicon (bp)	References
16S	8F	AGAGTTTGATCCTGGCTCAG	55	Small-subunit rRNA	1500	Weisburg *et al*., 1991
	1492R	TACGGHTACCTTGTTACGACTT				
*gyrB*	Up-1G-	YGCSGGCGGYAAGTTCGA	60	DNA gyrase subunit B	1208	Mavrodi *et al*., 2010
	UP-2G-	CCRTCGACGTCVGCRTCGGT				
	gyrBch1	CGCYGGCGGTAAGTTCGA	57	DNA gyrase subunit B	1208	This study
	gyrBch2	CCGTCGACGTCRGCGTCGGT				
*rpoD*	PsEG30F	ATYGAAATCGCCAARCG	60	Sigma 70 factor of RNA polymerase	760	Mulet *et al*., 2009
	PsEG790R	CGGTTGATKTCCTTGA				
*phl*D	B2BF	ACCCACCGCAGCATCGTTTATGAGC	66.5	2,4-DAPG-specific type III PKS	629	McSpadden Gardener *et al*., 2001
	BPR4	CCGCCGGTATGGAAGATGAAAAAGTC				
*phz*F	Ps_ up 1	ATCTTCACCCCGGTCAACG	57	Phenazine biosynthesis enzyme	427	Mavrodi *et al*., 2010
	Ps_low 1	CCRTAGGCCGGTGAGAAC				
*prn*D	PRND1	GGGCGGGCCGTGGTGATGGA	65	Pyrrolnitrin biosynthesis enzyme	786	De Souza and Raaijmakers, 2003
	PRND2	YCCCGCSGCCTGYCTGGTCTG				
*plt*C	PLTC1	AACAGATCGCCCCGGTACAGAACG	58	Type I PKS	438	De Souza and Raaijmakers, 2003
	PLTC2	AGGCCCGGACACTCAAGAAACTCG				
*acdS*	F1936f-tail	GCTCCTACTCTGTCACCTATCGHGAMGACTGCAAYWSYGGC	50	ACC deaminase- encoding gene	792	This study
	F1938r-tail	CTGTCGCTCTGGCTGTCATCATVCCVTGCATBGAYTT				
	Tail1	GCTCCTACTCTGTCACCTATC	60	See footnote		Nikolic *et al*., 2011
	Tail2	CTGTCGCTCTGGCTGTC				

The Tail1/Tail2 primer set was used for direct sequencing of *acdS* amplicons generated with primers F1936f-tail/F1938r-tail.

### Sequence and phylogenetic analysis

Sequence data were assembled and analysed using Geneious Pro v. 7.1.5 (Biomatters, Auckland, New Zealand). The 16S rDNA similarity searches and taxa assignments were carried out using the Classifier and SeqMatch tools available through the Ribosomal Database Project (http://rdp.cme.msu.edu). For antibiotic, ACC deaminase, *gyrB* and *rpoD* gene sequences, database searches for similar DNA and protein sequences were carried out with NCBI's BLAST network service (http://www.ncbi.nlm.nih.gov/BLAST). Sequences were aligned and neighbour-joining phylogenies were inferred using the Molecular Evolutionary Genetics Analysis software (MEGA) v. 5.2.2 (Tamura *et al*., 2007). DNA and protein distances were corrected by using the Kimura two-parameter (Kimura, 1980) and Jones-Taylor-Thornton (Jones *et al*., 1992) models of evolution, respectively, and bootstrap re-sampling with 1000 replicates was used to assess the reproducibility of clades in the inferred phylogenies.

### Preparation of barley and oat grain inoculum

Inocula of *G. graminis* var. *tritici* and *R*. *solani* AG-8 were prepared as described by Yang and colleagues (2011). Briefly, oat or barley grains (250 g) and water (350 ml) were combined in a flask and autoclaved on each of two consecutive days. Sterilized grains were inoculated with pieces of agar cut from ^1^/_5_ × PDA plates of *R. solani* or *G. graminis* var. *tritici*. After 21 days at room temperature, the colonized grains were tested for contamination, dried under a stream of sterile air and stored at 4°C. Prior to use, inoculum was fragmented and sieved into sizes, with particles of 0.25–0.5 mm added to soil.

### Bacterial treatment of wheat seed

For biocontrol studies, seeds were coated with bacteria by methods similar to those of Yang and colleagues (2011). Strains were inoculated onto plates of KMB or TSA and incubated at room temperature for 24–48 h. Bacteria were scraped into 1.0 ml H_2_O, washed by centrifugation (twice for 3 min at 14 000 r.p.m.) and then suspended in H_2_O. The concentration of the cell suspension was adjusted based on optical density (600 nm), and an aliquot was mixed with a 2% solution of methylcellulose (MC) and deionized H_2_O. Wheat (130 seeds) was added into the mixture, shaken for 3 min and seeds were dried under a stream of sterile air. To determine the final cfus per seed, 10 seeds were placed in a tube with 10 ml of sterile water, vortexed (1 min) and sonicated (1 min). Population size as determined by the end-point dilution assay ranged from 10^4^ to 10^7^ cfu seed^−1^.

### Biocontrol activity of rhizobacteria

The biocontrol activity of strains was determined by several approaches, and against both Rhizoctonia root rot and take-all, in autoclaved, pasteurized (60°C, 30 min) and raw soil. For pot experiments, soil from a farm at Huazhong Agricultural University, Wuhan, China, was sieved, autoclaved and amended with barley grain inoculum of *R. solani* (1%, w/w). Each plastic pot (10 cm deep × 6 cm wide) was filled with a 5 cm-thick layer of sterile vermiculite followed by 8 g of infested soil. Four treated wheat seeds (cv. Yangmai 158) were sown on the surface of the soil and covered with a 1.5 cm layer of sterile vermiculite. Controls included: control 1 (CK1), soil not amended with inoculum and sown to non-treated seeds; control 2 (CK2), soil amended with inoculum and sown to seed coated with MC; and control 3 (CK3), soil amended with inoculum and sown to non-treated seed. Each pot was given 10 ml of 2.5 mg ml^−1^ metalaxyl (Syngenta Wilmington, DE, USA) to prevent Pythium damping-off. Pots were covered with a plastic sheet to reduce evaporation, incubated at room temperature (22°C) overnight and then transferred to a greenhouse (17 ± 5°C). The plastic was removed when the tips of the shoots were visible. Each pot was watered twice weekly with water and once with ^1^/_3_ × Hoagland's solution (macro-elements only). Treatments were replicated five times and arranged in a randomized complete block design; each pot served as a replicate. After 3 to 4 weeks, the seedlings were removed from the pots, washed free of soil, and the plants were evaluated for disease severity on a scale of 0 to 8, where 0 = no lesions evident and 8 = seedling dead or nearly so (Huang *et al*., 2004). The length of the seedling shoot also was measured.

Biocontrol studies of Rhizoctonia root rot and take-all were also conducted using the tube assay as described by Yang and colleagues (2011) with pasteurized or raw Shano sandy loam (Quincy virgin) from a non-cropped site near Quincy, WA, USA. Plastic tubes (2.5 cm diameter, 16.5 cm long) with a hole in the bottom were hung in plastic racks (200 per rack). Each tube had a cotton ball placed in the bottom, followed by a 6.5 cm-thick column of sterile vermiculite, and then soil (10 g), with or without inoculum of *R. solani* at 1.0 % (w/w) or *G. graminis* var. *tritici* at 0.7% (w/w). A 1.5 cm-thick layer of vermiculite was placed over the infested soil, followed by three bacteria-treated wheat seeds (cv. Louise) and finally another 1.5 cm-thick topping of vermiculite. Each tube then received 10 ml of water with metalaxyl. Racks of tubes were covered with plastic sheets until shoots emerged and were incubated in a growth chamber (15–18°C; dark/light cycle 12 h). Each cone was watered twice weekly with water and once weekly with ^1^/_3_ × Hoagland's solution. Seedlings were harvested after 3–4 weeks and washed under a stream of water. The severity of Rhizoctonia root rot and take-all was evaluated on a scale of 0–8 (Yang *et al*., 2011).

### Detection of IAA

IAA production was determined colorimetrically (Patten and Glick, 2002; Egamberdieva *et al*., 2008). Strains were incubated with shaking for 48 h at 28°C in KMB broth alone or supplemented with 500 μg per millilitre of tryptophan. Cultures were centrifuged at 13 000 r.p.m. for 10 min and a 1 ml aliquot of the supernatant was mixed vigorously with 4 ml of Salkowski reagent. The mixture was incubated at room temperature for 25 min and the absorbance of the pink colour was measured spectrophotometrically at 540 nm. The concentration of IAA was determined by using a calibration curve of pure IAA (0, 20, 40, 60, 80 and 100 μg ml^−1^) as a standard. Readings of < 1 μg ml^−1^ per OD_600_ unit were considered as negative for IAA production.

### Growth on ACC as a sole nitrogen source

Strains were tested for ability to grow in a defined medium supplemented with ACC as a sole nitrogen source as described by Penrose and Glick (2003). Strains were first cultured in 3 ml of sterile DF salts minimal medium (pH 7.2): per litre, KH_2_PO_4,_ 4.0 g; Na_2_HPO_4,_ 6.0 g; MgSO_4_ 7H_2_O, 0.2 g; glucose, 2.0 g; gluconic acid, 2.0 g; citric acid, 2.0 g; trace elements (FeSO_4_ 7H_2_O, 1 mg; H_3_BO_3_, 10 μg; MnSO_4_ H_2_O, 11.19 μg; ZnSO_4_ 7H_2_O, 124.6 μg; CuSO_4_ 5H_2_O, 78.22 μg; MoO_3,_ 10 μg); and (NH_4_)_2_ SO_4,_ 2.0 g as a nitrogen source. After 72 h of shaking at 28^o^C, a 5 μl aliquot was transferred to 3 ml sterile DF salts medium with 3.0 mM ACC instead of (NH_4_)_2_ SO_4_ as the source of nitrogen. *Pseudomonas brassicacearum* Q8r1-96 was used as a positive control.

### Canola root elongation assay

A gnotobiotic canola root elongation assay was used to determine the ability of strains to promote growth (Egamberdieva *et al*., 2008). An aliquot of an overnight culture was centrifuged, the cell pellet was washed twice and suspended in 1 ml of sterile 0.03 M MgSO_4._ The suspension was adjusted to an OD_600_ of 0.15. Surface-sterilized spring canola seeds (cv. InVigor 8440) were soaked for 1 h in 700 μl of a bacterial suspension. Depending on the isolate, the concentration ranged from 10^4^ to 10^6^ cfu seed^−1^. Growth pouches were made using 1-gallon Ziploc plastic bags and sterile germination paper soaked with 50 ml of ^1^/_5_ × Hoagland's solution. Twenty-five seeds of a single bacterial treatment were distributed along the germination paper and the paper was placed inside of the bag. Individual bags were then hung vertically, covered with aluminium foil for 1–2 days and incubated at 20°C in a dark/light cycle of 12 h. The length of the primary roots was measured 5 days after sowing. Roots of seeds that failed to germinate 2 days after sowing were not measured. This study was conducted five times with three representative experiments shown.

### Tolerance of bacteria to abiotic stress

The tolerance of selected strains to copper, cadmium and NaCl was determined in Luria–Bertani (LB) broth amended with CuCl_2_ to give concentrations from 3 to 6 mM; CdCl_2_ to give concentrations from 0.4 to 2.0 mM; and NaCl to give concentrations from 0% to 8%. Each well of a 96-well microplate was filled with 200 μl of LB supplemented with the heavy metal or salt solutions and inoculated with 2 μl of an overnight culture of each isolate (OD_600_ = 0.1). Each isolate at each concentration of heavy metal or salt was replicated eight times. The microplates were incubated at room temperature in the dark for 72 h and assayed spectrophotometrically at 600 nm using a model 680 microplate reader (Bio-Rad). An OD_600_ > 0.1 was scored as positive for growth. The MIC was defined as the lowest concentration that completely inhibited the growth of the isolate.

### Statistical analysis

Comparisons of means of root disease ratings were performed using standard analysis of variance (ANOVA) followed by Kruskal–Wallis all pairwise comparisons (*P* ≤ 0.05) (Statistix 8.1, Analytical Software, Tallahassee, FL, USA). Mean comparisons of plant height and root length among treatments were determined by ANOVA followed by using either the Fisher's protected least significant difference test (*P* = 0.05) or the Kruskal–Wallis all pairwise test (*P* = 0.05). Bacterial population sizes were log transformed before analysis.

### Accession numbers

Nucleotide sequences were deposited in GeneBank under the following accession numbers: *pltC,* KC357589 through KC357592; *prnD,* KC693004 through KC693007; *phlD,* KC693000 through KC693003; *acdS,* KC430107 through KC430112; *gyrB*, KM030038 through KM030046; *rpoD*, KM030047 through KM030055; 16S rDNA, KM030056 through KM030064. The *phzF* sequence of strain KY5406 was deposited under accession number KC572125.

## Supporting Information

**Fig. S1.** Neighbour-joining phylogeny inferred from concatenated housekeeping genes 16S, *gyrB* and *rpoD*. The concatenated housekeeping gene data set contained 2911 characters. Evolutionary distances were estimated using the Kimura two-parameter model of nucleotide substitution. Indels were ignored in the analysis and reproducibility of clades was assessed by bootstrap resampling with 1000 pseudoreplicates. The branch lengths are proportional to the amount of evolutionary change. The scale bars indicate substitution per site. Subgroups within the *P*. fluorescens lineage are defined according to Mulet and colleagues (2010). **Fig. S2.** Neighbour-joining phylogeny inferred from data for aligned 578–bp fragments of *phlD* sequences of fluorescent *Pseudomonas* spp. Indels were ignored in the analysis, and evolutionary distances were estimated using the Kimura two-parameter model of nucleotide substitution. Bootstrap values greater than 60% are indicated at the nodes, and the branch lengths are proportional to the amount of evolutionary change. Clades indicated by capital letters correspond to BOX-PCR genotypes of 2,4-diacetylphloroglucinol-producing *Pseudomonas* spp. as defined by De La Fuente and colleagues (2006). **Fig. S3.** Neighbour-joining phylogeny inferred from data for aligned 390–bp fragments of *pltC* sequences of fluorescent *Pseudomonas* spp. Indels were ignored in the analysis, and evolutionary distances were estimated using the Kimura two-parameter model of nucleotide substitution. Bootstrap values greater than 60% are indicated at the nodes, and the branch lengths are proportional to the amount of evolutionary change. **Fig. S4.** Neighbour-joining phylogeny inferred from data for aligned 745–bp fragments of *prnD* sequences of pyrrolnitrin-producing species. Indels were ignored in the analysis, and evolutionary distances were estimated using the Kimura two-parameter model of nucleotide substitution. Bootstrap values greater than 60% are indicated at the nodes, and the branch lengths are proportional to the amount of evolutionary change. Clades C1 through C2.4 correspond to distinct groups of pyrrolnitrin-producing bacteria as defined by Costa and colleagues (2009). **Fig. S5.** Neighbour-joining phylogeny inferred from data for aligned 391–bp fragments of *phzF* sequences of fluorescent *Pseudomonas* spp. Indels were ignored in the analysis, and evolutionary distances were estimated using the Kimura two-parameter model of nucleotide substitution. Bootstrap values greater than 60% are indicated at the nodes, and the branch lengths are proportional to the amount of evolutionary change. Clades A1 through A5 correspond to distinct groups of phenazine-producing bacteria as defined by Mavrodi and colleagues (2010). **Fig. S6.** Neighbour-joining phylogeny inferred from data for aligned 714–bp fragments of *acdS* sequences of ACC-deaminase-producing bacteria. Indels were ignored in the analysis, and evolutionary distances were estimated using the Kimura two-parameter model of nucleotide substitution. Bootstrap values greater than 60% are indicated at the nodes, and the branch lengths are proportional to the amount of evolutionary change. Clades A through C correspond to distinct groups of ACC-deaminase-producing bacteria as defined by Blaha and colleagues (2006).
