# Supplementary material for: Biocontrol and plant growth-promoting activity of rhizobacteria from Chinese fields with contaminated soils
Source: Microb Biotechnol. 2014 Sep 15;8(3):404–18. doi: 10.1111/1751-7915.12158 (PMC4408174; doi:10.1111/1751-7915.12158)
Supplement: Supplementary file 1 [file mbt20008-0404-sd1.zip › SUPPLEMENTARY REFERENCES.docx]

SUPPLEMENTARY REFERENCES

Blaha, D., Pringet-Combaret, C., Mirza, M. S., and Moënne-Loccoz, Y. (2006) Phylogeny of the 1-aminocyclopropane-1-carboxylic acid deaminase-encoding gene *acdS* in phytobeneficial and pathogenic Proteobacteria and relation with strain biogeography. FEMS Microbiol Ecol 56:455-470.

Costa, R., Van Aarle, I. M., Mendes, R., and Van Elsas, J. D. (2009) Genomics of pyrrolnitrin biosynthetic loci: evidence for conservation and whole-operon mobility within Gram-negative bacteria. Environ Microbiol **11:** 159–175.

De la Fuente, L., **Mavrodi, D. V., Landa, B. B., Thomashow, L. S., and Weller, D. M. (2006)** phlD-based genetic diversity and detection of genotypes of 2,4-diacetylphloroglucinol-producing Pseudomonas fluorescens. FEMS Microbiol Ecol **56:**64-78.

Mavrodi, D. V., Peever, T. L., Mavrodi, O. V., Parejko, J. A., Raaijmakers, J. M., Lemanceau, P., Mazurier, S., Heide, L., Blankenfeldt, W., Weller, D. M., and Thomashow, L. S. (2010) Diversity and evolution of the phenazine biosynthesis pathway. Appl.EnvironMicrobiol **76:** 866–879.

Mulet, M., Lalucat, J., and García-Valdés, E. (2010) DNA sequence-based analysis of the *Pseudomonas*  species. *Environ Microbiol* **12:** 1513-1530.
