# Supplementary figures and images for: Biocontrol and plant growth-promoting activity of rhizobacteria from Chinese fields with contaminated soils
Source: Microb Biotechnol. 2014 Sep 15;8(3):404–18. doi: 10.1111/1751-7915.12158 (PMC4408174; doi:10.1111/1751-7915.12158)

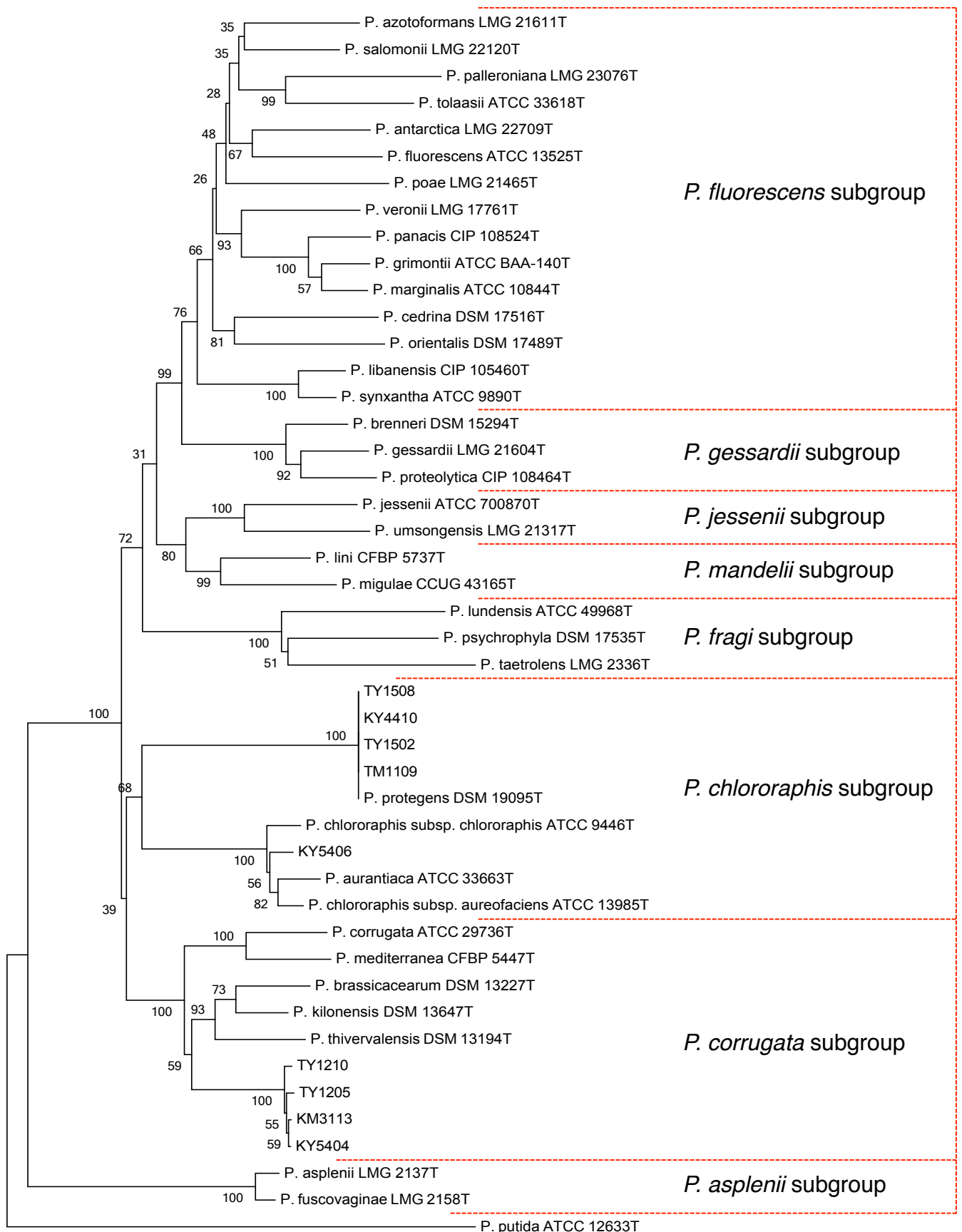

Supplement: Supplementary file 1 [file mbt20008-0404-sd1.zip › Fig_S1.pdf]

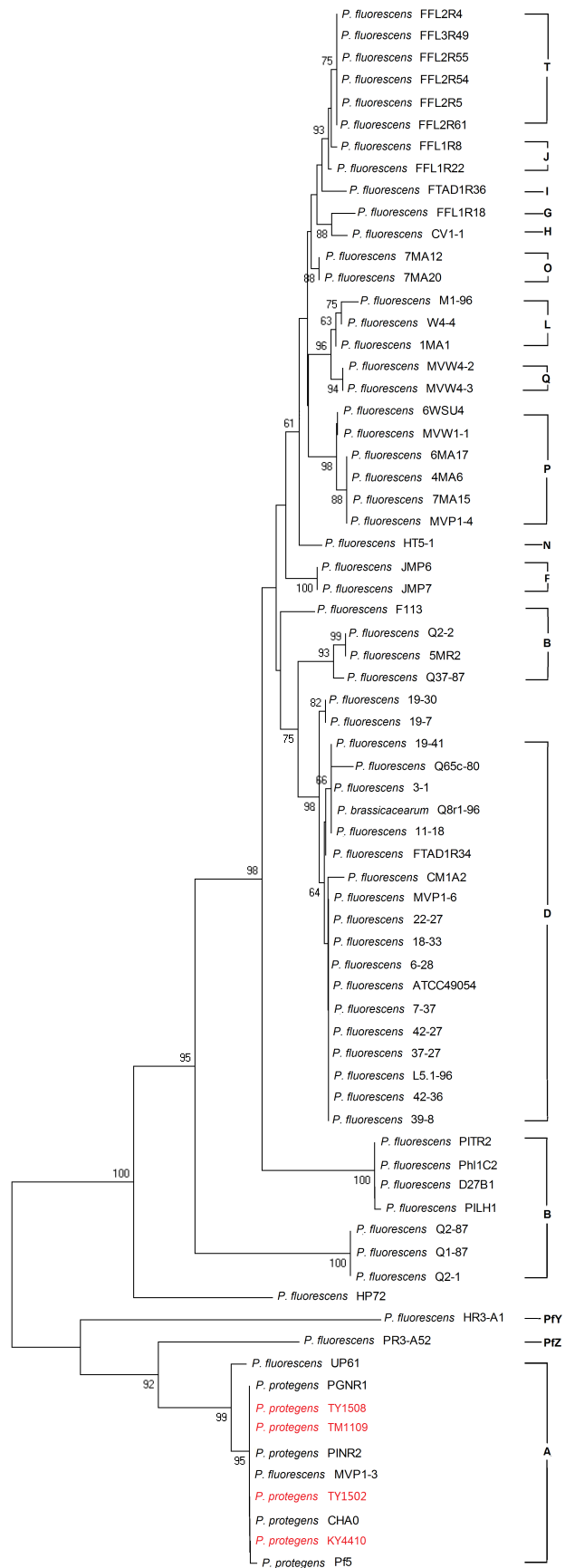

Supplement: Supplementary file 1 [file mbt20008-0404-sd1.zip › Fig_S2.pdf]

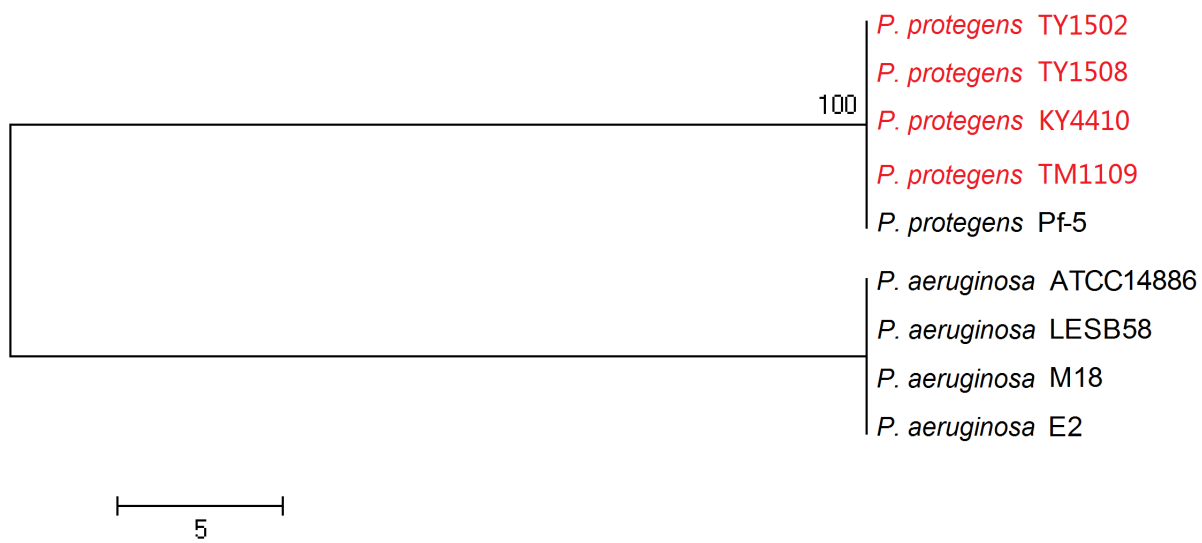

Figure S3

Supplement: Supplementary file 1 [file mbt20008-0404-sd1.zip › Fig_S3.pdf]

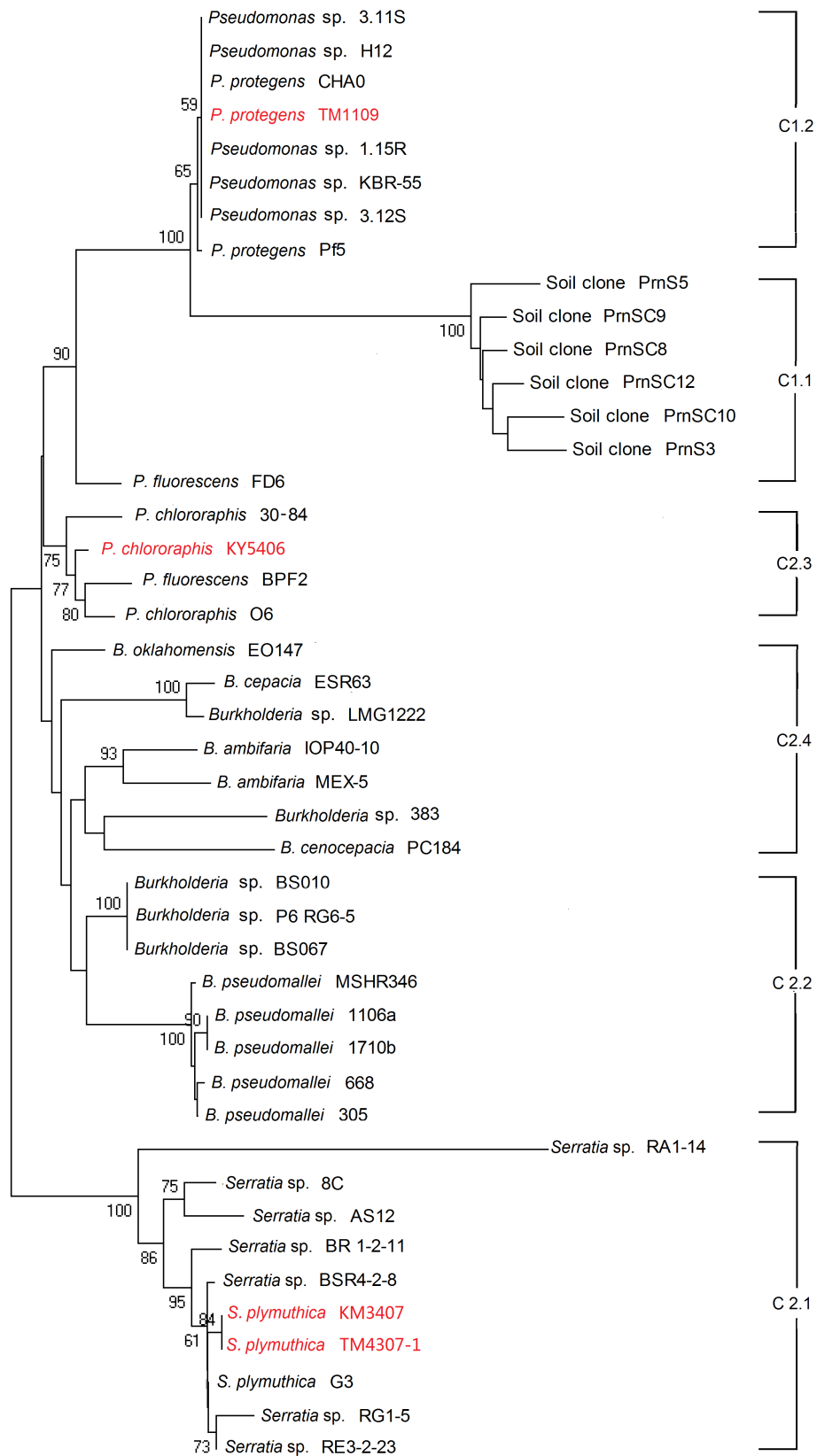

0.01

Supplement: Supplementary file 1 [file mbt20008-0404-sd1.zip › Fig_S4.pdf]

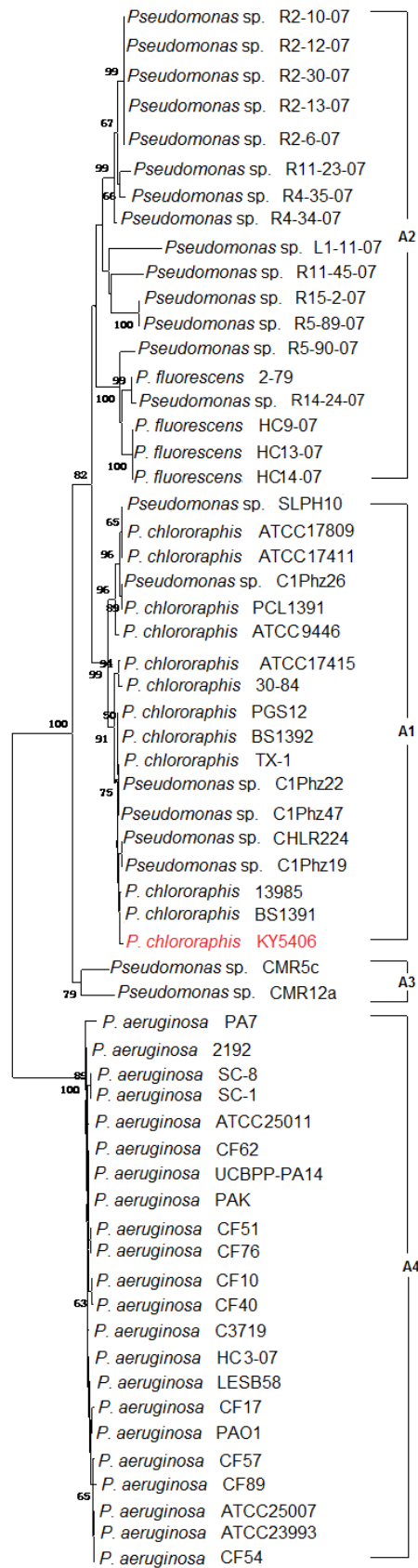

Supplement: Supplementary file 1 [file mbt20008-0404-sd1.zip › Fig_S5.pdf]

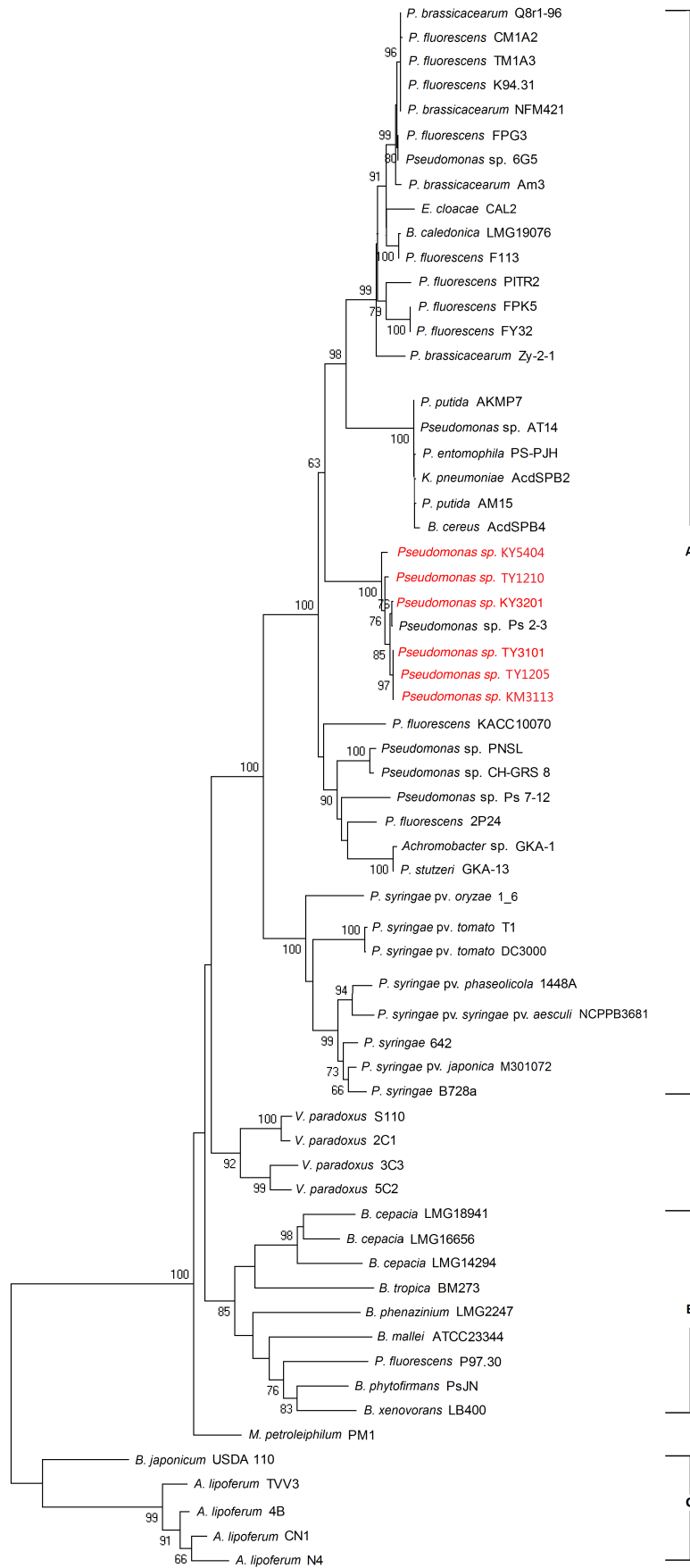

Supplement: Supplementary file 1 [file mbt20008-0404-sd1.zip › Fig_S6.pdf]
